# Roles of a Cryptochrome in Carbon Fixation and Sucrose Metabolism in the Liverwort *Marchantia polymorpha*

**DOI:** 10.3390/cells10123387

**Published:** 2021-12-01

**Authors:** Tianhong Li, Li Zhang, Shengzhong Su, Sudi Li, Junchuan Zhang, Zhenming Yang, Zecheng Zuo

**Affiliations:** Jilin Province Engineering Laboratory of Plant Genetic Improvement, College of Plant Science, Jilin University, Changchun 130062, China; litianhong2011@hotmail.com (T.L.); zhang_li18@mails.jlu.edu.cn (L.Z.); sushengzhong@jlu.edu.cn (S.S.); lisd19@mails.jlu.edu.cn (S.L.); jczhang_gray@163.com (J.Z.)

**Keywords:** *Marchantia polymorpha*, cryptochromes, carbon fixation, sucrose metabolism, asymmetric growth of thallus

## Abstract

In vascular plants, cryptochromes acting as blue-light photoreceptors have various functions to adapt plants to the fluctuating light conditions on land, while the roles of cryptochromes in bryophytes have been rarely reported. In this study, we investigated functions of a single-copy ortholog of cryptochrome (*MpCRY*) in the liverwort *Marchantia polymorpha.* Knock-out of Mp*CRY* showed that a large number of the mutant plants exhibited asymmetric growth of thalli under blue light. Transcriptome analyses indicated that Mp*CRY* is mainly involved in photosynthesis and sugar metabolism. Further physiological analysis showed that Mp*cry* mutant exhibited a reduction in CO_2_ uptake and sucrose metabolism. In addition, exogenous application of sucrose or glucose partially restored the symmetrical growth of the Mp*cry* mutant thalli. Together, these results suggest that Mp*CRY* is involved in the symmetrical growth of thallus and the regulation of carbon fixation and sucrose metabolism in *M. polymorpha*.

## 1. Introduction

The perception and response of organisms to blue light have been a focus of scientific interest [1], especially on cryptochrome (cry), one of the blue light receptors found in the evolutionary lineages of archaea, bacteria, plants and animals [1,2,3]. Cryptochromes from aquatic to terrestrial organisms retain fairly conservative functions and also generate relatively novel functions [4]. Cryptochromes from plants and animals are considered to have originated from two independent evolutionary events [2,4]. Cryptochromes in animals regulate the circadian clock and magnetoreception [5,6,7]. In vascular plants, cryptochromes have various functions to adapt to the fluctuating light conditions on land, including the regulation of the circadian clock, the hypocotyl elongation under blue light, photoperiodic flowering, cotyledon expansion, shade avoidance, and stomata opening [8,9,10,11,12,13].

Previous studies on bryophyte cryptochromes are limited to *Physcomitrium patens*. There are two cryptochromes in *P. patens*, *PpCRY1a* and *PpCRY1b*, which play the roles in induction of side branching of protonema and differentiation and growth of gametophore [14]. In addition, PpCRYs influence the daily expression profiles of the orthologs of sigma factors (*PpSigs*) and RNA polymerase subunit alpha (*PpRpoA*). PpCRYs also regulate the transcription of Squamosa promoter binding protein orthologs (*PpSBP1* and *PpSBP4*) to induce side branch formation under blue light [15,16]. These studies indicate that cryptochromes are an important factor in the transduction of blue light signal in *P. patens*. However, the specific mechanism of cryptochromes affecting the growth and development of bryophytes still remains to be analyzed.

The liverwort *Marchantia polymorpha* has recently been established as a model plant species. It has a life cycle whose dominant form is haploid, and its genome shows a low genetic redundancy [17,18]. In addition, its genetic transformation and gene targeting systems have been established [19]. Previous studies have shown the functions of the single-copy phytochrome ortholog MpPHY, the ultraviolet-B photoreceptor MpUVR8, and the blue light receptors MpPHOT and MpFKF in *M. polymorpha* [20,21,22,23,24]. However, although present in the genome [18], the function of MpCRY has never been reported.

To study the function of MpCRY, we obtained Mp*cry* knock-out mutants using CRISPR/Cas9-mediated genome editing technology [25]. Interestingly, we found that Mp*CRY* is required for symmetrical growth of thallus under continuous blue light. We also found that MpCRY is involved in the regulation of photosynthesis and sucrose metabolism. Together, these results provide new insights into the function of MpCRY during growth and development of *M. polymorpha*.

## 2. Materials and Methods

### 2.1. Plant Materials and Growth Conditions

Male and female *M. polymorpha* accessions, Takaragaike-1 (Tak-1) and Takaragaike 2 (Tak-2) [26], were cultured aseptically on half-strength Gamborg’s B5 medium [27] under continuous white light of 50 to 60 μmol photon m^−2^ s^−1^ at 22 °C. F1 spores were obtained by crossing Tak-2 and Tak-1.

### 2.2. Phylogenetic Tree Analysis

For the alignment of amino acid sequences, we used the MUSCLE program in Geneious software (Biomatters, Auckland, New Zealand, version 8.1.3) [28]. After sequence alignment, sequence gap and sequences at both ends were manually removed, and a conserved region was retained to calculate evolutionary distance. A phylogenetic tree was built by the online program PhyML 3.0 (www.atgc-montpellier.fr/phyml/ (accessed on 21 March 2020)) [29] with JTT model and four substitution rate categories. Its tree searching was started from the aBioNJ tree, and the tree was optimized with Subtree Pruning and Regrafting (SPR) topological moves. For statistical analysis of the constructed phylogenetic tree, bootstrapping was carried out by resampling trees 1000 times. The beautification of the resulting tree was done in Geneious software. Amino acid sequences of *Amborella trichopoda*, *Selaginella moellendorffii*, *Physcomitrella patens*, *Marchantia polymorpha*, *Chlamydomonas reinhardtii*, and *Ostreococcus lucimarinus* are from https://phytozome-next.jgi.doe.gov (accessed on 8 December 2015). Amino acid sequences of *Arabidopsis thaliana* are from https://www.arabidopsis.org (accessed on 8 December 2015). Amino acid sequences of *Klebsormidium flaccidum* are from http://www.plantmorphogenesis.bio.titech.ac.jp/~algae_genome_project/klebsormidium/ (accessed on 8 December 2015).

### 2.3. Generation of Transgenic Lines

To generate the Mp*cry* knockout mutant, we used the CRISPR/Cas9 genome editing system [30]. A guide RNA (gRNA) target sequence was designed in the 4th exon of the Mp*CRY* gene. The annealed oligonucleotides (MpCRY-gRNA-F/MpCRY-gRNA-R) of the gRNA sequence was cloned into the BsaI site of pMpGE_En03 [31]. Using the Gateway LR reaction (Thermo Fisher Scientific, Torrance, CA, USA), the MpCRY gRNA expression cassette was transferred to the pMpGE011 vector [31] to generate pMpGE011_MpCRY. As described previously [26], *Agrobacterium*-mediated transformation of F1 spores was performed. In order to identify gene knockout mutants, crude genomic DNA was extracted from the transformed thalli, and the region targeted by the gRNA sequence was amplified using primers MpCRY-check-f/MpCRY-check-r. The PCR products were directly sequenced using BigDye Terminator v3.1 (Thermo Fisher Scientific).

In order to obtain the MpCRY strain overexpressing the Citrine marker, the CDS sequence of Mp*CRY* without the stop codon from wild-type genomic DNA, which was subcloned into pENTR/D-TOPO vector (Thermo Fisher Scientific). The cloned sequence was then transferred to the pMpGWB306 vector [32]. Regenerated thalli were used for *Agrobacterium*-mediated genetic transformation [33]. To obtain the overexpression lines of MpCRY-Tdtomato, the coding sequence (CDS) of MpCRY without the stop codon was amplified and cloned into pENTR/D-TOPO vector (Thermo Fisher Scientific). Then the cloned sequence was transferred to the destination vector pMpGWB130 [32] to generate *_pro_35S::MpCRY-Tdtomato* binary vector, which was transformed into WT (Tak-1) thalli. The sequences of all primers used in transgenic plant generation are listed in Table S2.

### 2.4. RNA Extraction and Transcriptome Sequencing

Three biological repeats of total RNA were extracted from three different groups of 2-week-old thalli grown from gemmae using RNAprep pure Plant Kit (TIANGEN BIOTECH, Beijing, China). RNA purity and concentration were tested using NanoDrop 2000 spectrophotometer (Thermo Fisher Scientific) and Agilent 2100 RNA 6000 Nano kit (Agilent Technologies). RNA sequencing analysis (RNA-seq) was performed by Beijing Berry Genomics Co., Ltd. (Berry Genomics Beijing, Beijing, China). RNA molecules were isolated using the poly-A selection protocol to enrich for polyadenylated transcripts. Illumina NovaSeq 6000 was applied paired-end sequencing for RNA-Seq.

### 2.5. Quantification of Gene Expression Levels in Transcriptome Sequencing

Sequencing reads were filtered by ht2-filter to remove low-quality reads [34], and then mapped to the genomic sequence of *Marchantia polymorpha* ver 3.1 [18] using TopHat2 with default parameters, i.e., the anchor length of reads was set up as 8 and the maximum number of mismatched bases that can appear in the anchor region was set up as 0 [35]. The relative abundance of mRNA was normalized and presented as fragments per kilobase of transcript per million mapped reads (FPKM) [36].

### 2.6. Person Correlation Coefficients Analysis

Person correlation coefficients were calculated by the “chart.Correlation” function of the PerformanceAnalytics package in R.

### 2.7. Principal Component Analysis

Contribution ratios were calculated by the “PCA” function of the FactoMineR package in R. The data frame used in this calculation was composed by the FPKMs of different samples.

### 2.8. Differential Expression Analysis

Differential expression analysis was performed using edgeR package with default parameters [37]. Genes showing larger than 1.5-fold change of expression compared to control with *p*-value < 0.05 were considered as differentially expressed genes (DEGs). Venn diagram was created using the ‘VennDiagram’ R package (version 1.6.20).

### 2.9. GO Enrichment Analysis of Different Response Genes (DRGs)

Gene Ontology (GO) enrichment analysis of DRGs was implemented by Blast2GO (Valencia, Spain, version 2.5.0) [38]. GO terms were enriched by Blast2GO and we chose GO terms of biological functions (BP) with a *p*-value less than 0.05 calculated by the hypergeometric test.

### 2.10. Processing Subcellar Images

Leica TCS SP8X confocal microscope was used to observe the gemmalings. A 514 nm laser was used for excitation of the fluorescent protein Citrine, and the emitted fluorescence with wavelength of 520–550 nm was captured. To gain complete picture of plant sample, 111 pictures were captured per 0.686 μm along the z axis. The series of images were analyzed and merged using LAS X (Leica) software (Leica Microsystems, Wetzlar, Germany). To magnify the part of plant cells, 48.94 μm × 48.94 μm ROI (region of interest) was selected, and only one image along the z axis under the same laser condition was captured. W 552 nm laser was used for excitation of the fluorescent protein Tdtomato. 10 μg/mL Hoechst 33342 (Sigma, 14533) was used to stain the nucleus [39]. D 405 nm laser was used for excitation of the Hoechst 33342. Calcein acetoxymethyl (AM) was used to stain the whole cell including the nucleus and cytoplasm [40]. W 488 laser was used for excitation of the Calcein AM. Images were analyzed and merged using LAS X (Leica) software.

### 2.11. Quantitative Real-Time PCR (RT-PCR) Analysis

cDNA was synthesized from 1 µg total RNA using SuperScript first-strand cDNA synthesis system (Thermo Fisher Scientific). SYBR Premix Ex Taq^TM^ II (Tli RNaseH Plus) (TaKaRa-Bio, Kyoto, Japan) was used for quantitative reverse transcription PCR (qRT-PCR) reaction, using the Mx3000P^TM^ Real-Time PCR System (Stratagene). Mp*ACT* expression was measured for the internal control [41]. The sequences of all primers used in qPCR are listed in Table S2.

### 2.12. Measurement of Chlorophyll Content

Thalli grown under blue light were harvested in liquid nitrogen (LN) and ground into fine powder. The sample powder was weighed, mixed with 1 mL of cool 80% *v*/*v* acetone, and kept in the dark at 4 °C overnight. After centrifugation, supernatant was used to measure OD at 645 nm and 663 nm with a spectrometer. Chlorophyll content was calculated using the following equation: Chlorophyll (mg/g) = (8.02 × OD663 + 20.20 × OD645) × V/FW, where V is the volume of the extract (in milliliters) and FW is the fresh weight of the sample powder (in milligrams).

### 2.13. Photosynthetic Parameter Analysis

For the measurement of Fv/Fm, 14-day-old thalli were dark adapted for 30 min and then were detected by IMAGING PAM (WALZ). A portable photosynthesis system (LI-6400, Li-Cor) was used to measure total conductance to CO_2_ in the 14-day-old thalli of WT (Tak-1) and Mpcry mutant plants. All measurements were performed at 22 °C and a relative humidity of 60%. The CO_2_ level and the photosynthetic photon flux density in the chamber was set to 400 μmol mol^−1^ and 1000 μmol m^−2^ s^−1^, respectively.

### 2.14. Quantification of Starch, Sucrose, Glucose

Plants were harvested into LN and finely ground, and 0.1 g of power was used for each of the following analyses: The starch levels were determined by Amylum Content Assay Kit (Sangon Biotech, Shanghai, China, D799325-0050). The sucrose levels were determined by Plant Sucrose Content Assay Kit (Sangon Biotech, D799789-0050). The glucose levels were determined by Glucose Content Assay Kit (Sangon Biotech, Shanghai, China D799407-0050).

### 2.15. Quantification and Statistical Analysis

All data were statistically analysed by two-tailed Student’s *t*-test.

## 3. Results

### 3.1. The Sequence Conservation of MpCRY

**Figure 1:**
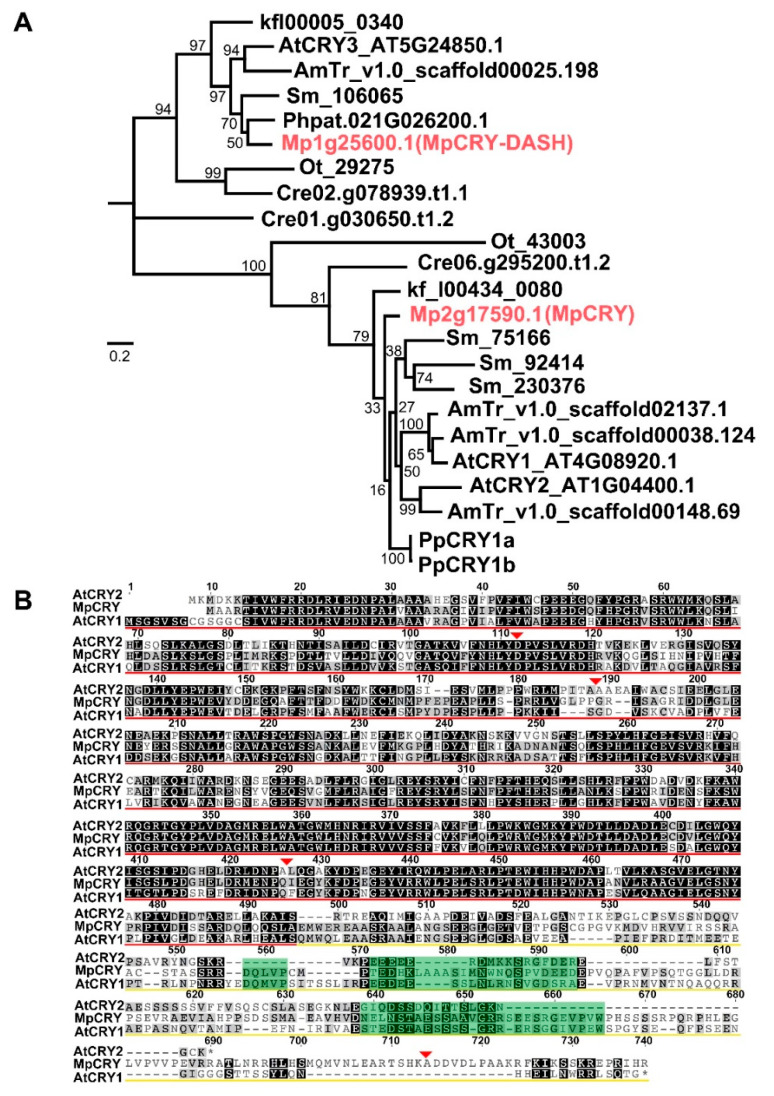
Phylogenetic analysis of cryptochromes from green algae and land plants. (**A**) Phylogenetic relationships of plant cryptochromes. The tree was constructed using 23 cryptochromes from various plant species. The *Ostreococcus lucimarinus* Ot_43003 sequence was used as the outgroup. Posterior probabilities are indicated at the nodes. At, Arabidopsis thaliana; *AmTr*, *Amborella trichopoda; Sm, Selaginella moellendorffii; Pp/Phpat, Physcomitrium patens*; *Mapoly*, *Marchantia polymorpha*; Kf, *Klebsormidium nitens*; Cre, *Chlamydomonas reinhardtii*; Ot, *Ostreococcus lucimarinus*. Bar indicates 0.1 substitutions per site. (**B**) Alignment of amino acid sequences of cryptochromes from *M. polymorpha* and *A. thaliana*. Identical and similar amino acid residues are highlighted with black and gray boxes, respectively. Red and yellow underlines indicate N-terminal PHR domain and CCE domain, respectively. Green background sequences indicate the DAS domain. Red inverted triangles indicate positions corresponding to intron insertion in the genes.* indicates the end point of the amino acid sequence.

Cryptochromes are phylogenetically classified into three distinctive groups, which are represented by plant cryptochromes, animal cryptochromes, and CRY-DASH (cryptochrome-Drosophila, Arabidopsis, Synechocystis, human), respectively [42]. Previous studies have indicated that CRY-DASH primarily plays a role in repairing cyclobutane pyrimidine dimmers, and plant cryptochromes are the major pathway for plant growth and development under blue light [42,43]. Our phylogenetic analysis suggested that the genome of *M. polymorpha* contains single orthologs of cryptochrome and CRY-DASH (Figure 1A). In this study, we focused on the function of MpCRY (Mp2g17590). The MpCRY gene possesses six exons and five introns, and encodes a protein of 715 amino acids containing a photolyase homologous region (PHR) domain at the N-terminus and a DQXVP-acidic-STAESSS (DAS) domain in the C-terminal cryptochrome C-terminal extension (CCE) domain, both of which are found in most plant cryptochromes [44] (Figure 1B). These results suggest that MpCRY has an evolutionarily conserved structure.

### 3.2. MpCRY Regulates the Thallus Symmetry of M. polymorpha under Blue Light

**Figure 2:**
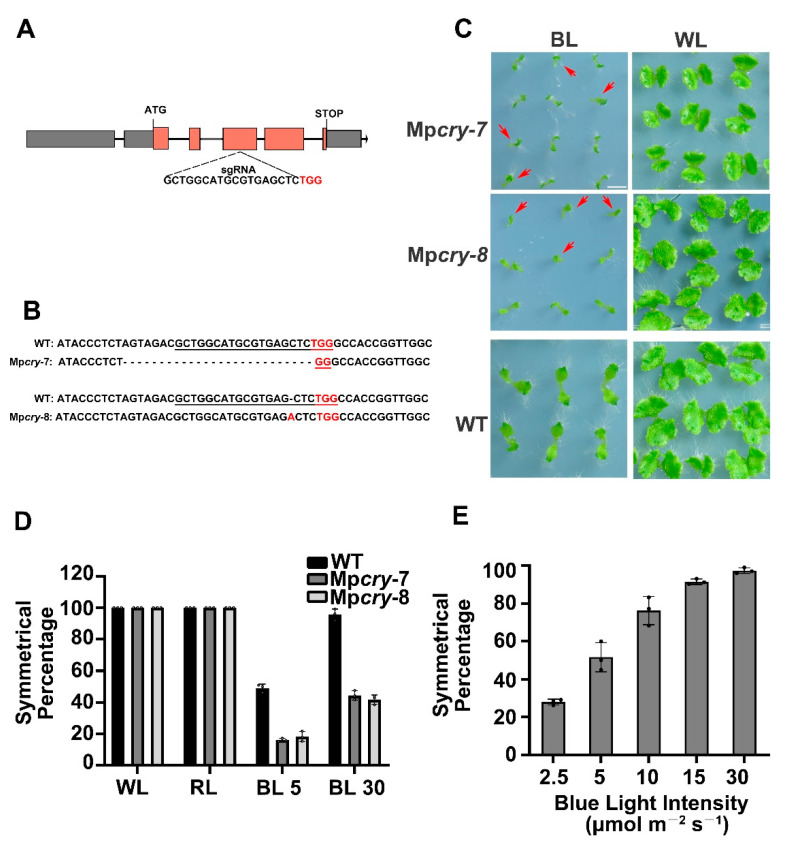
Growth of gemmalings of WT and Mp*cry* mutants under various BL conditions. (**A**) Schematic diagram of the structure of the MpCRY gene and the target sequence of CRISPR/Cas9 genome editing. Grey boxes indicate 5′ UTR and 3′ UTR. Orange boxes indicate CDS regions. Exons contain grey boxes and orange boxes. Black lines indicate introns. (**B**) Mp*cry* mutations detected by sequencing analysis. The PAM (protospacer adjacent motif) sequence is highlighted in red, and the target sequence is underlined in black. Dashes indicate deleted bases. (**C**) Photographs of gemmalings under BL or WL for 14 days. Bar = 5 mm. The red arrows represent individuals with asymmetric growth of thallus. (**D**) The symmetrical percentage represents the percentage of plants showing symmetric growth under the indicated light conditions. The gemmalings of WT, Mp*cry-7*, and Mp*cry-8* were grown under continuous WL (WL; 50 μmol m^−2^ s^−1^), continuous RL (RL; 30 μmol m^−2^ s^−1^) or continuous BL (BL; 30 μmol m^−2^ s^−1^) for 14 days, respectively. Data are presented as mean ± SD (*n* = 3 biological statistics). More than 100 gemmalings were used for one count. (**E**) The symmetrical percentage of WT thalli under continuous BL irradiation at various light intensities (2.5 to 30 μmol m^−2^ s^−1^). Data are presented as mean ± SD (*n* = 3 biological statistics). More than 100 gemmalings were used for one count.

To study physiological function of MpCRY, we used a CRISPR/Cas9 system to generate mutants [30], which were named Mp*cry-7* and Mp*cry-8* respectively (Figure 2A,B). Mp*cry-7* and Mp*cry-8* have a 26-bp deletion and a 1-bp insertion in the 4th exon of MpCRY, respectively (Figure 2A,B). To confirm both Mp*cry-7* and Mp*cry-8* are knock-out lines, we performed a qPCR assay to detected the mRNA levels of MpCRY in Mp*cry-7* and Mp*cry-8* mutants and found that the expression of MpCRY was abolished in both mutant lines (Figure S1A). These mutants were used in the subsequent experiments. It was reported that spores and gemmae of *M. polymorpha* did not germinate under dark conditions [45,46]. We first incubated the gemmae, vegetative propagules from gemmae gups on the thalli, from WT and Mp*cry* in darkness and then exposed to continuous white light (WL), red light (RL), and blue light (BL), for two weeks. Under WL, both of the gemmalings of WT and Mp*cry* grew into flattened thalli with symmetrical dichotomous branches (Figure S1B). With continuous illumination of RL, there was no significant growth differences between WT and Mp*cry*, and the size of the thalli was slightly smaller than that of thalli grown under WL (Figure S1B). In contrast, both of WT and Mp*cry* thalli grown under BL (30 μmol m^−2^ s^−1^) showed slender shape and oblique angle growth. Interestingly, 56% of Mp*cry-7* and 58.2% of Mp*cry-8* showed growth from only one side of the gemma (hereafter referred to as ‘asymmetric growth of thallus’) under BL, respectively, while more than 90% of WT plants showed growth from both two sides of the gemma (Figure 2C,D). This asymmetric thallus growth was likely caused by the asymmetric germination of gemmae (Figure S2). To explore the relationship between BL intensity and the asymmetric growth of thalli, we observed the thallus growth of WT exposed to different intensities of BL: The percentage of symmetrically grown thalli decreased by lowering BL intensity from 30 to 2.5 μmol m^−2^ s^−1^ in a dose-dependent manner (Figure 2E). We found that under BL of 5 μmol m^−2^ s^−1^ intensity, the percentage of symmetric growth in Mp*cry-7* and Mp*cry-8* was 16% and 18% respectively, significantly lower than that in WT (49%) (Figure 2D), suggesting that Mp*cry* mutants are less sensitive to BL. These results suggest that BL has a positive effect on the symmetric growth of thallus, and this event is promoted by MpCRY in *M. polymorpha*.

### 3.3. Transcriptome Analysis of Mpcry Mutants under Blue Light

**Figure 3:**
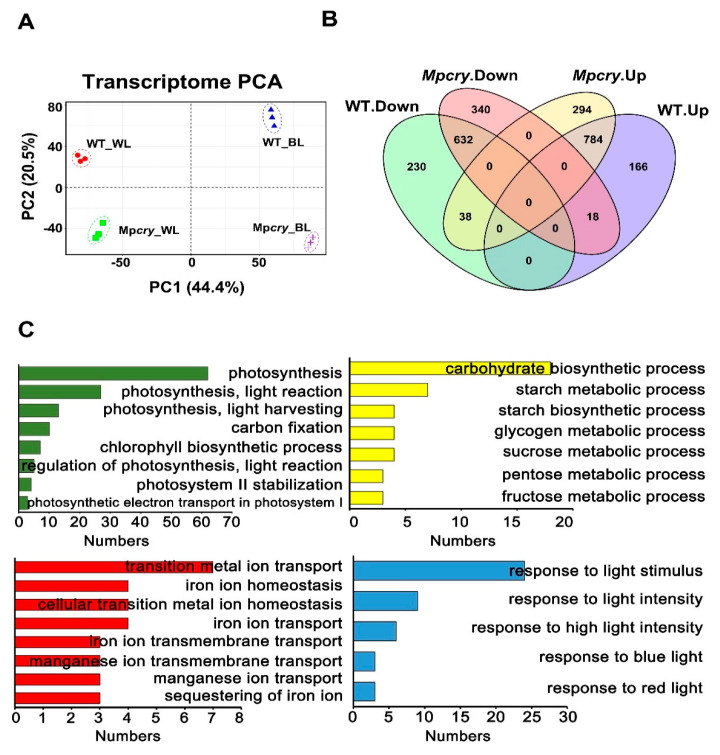
Differential response genes (DRGs) in WT and Mp*cry* mutant plants grown under BL and WL conditions and GO enrichment analysis. (**A**) Principal component analysis (PCA) of transcriptome changes of between WT and Mp*cry* under different light treatments. (**B**) Venn diagram showing overlaps of DEGs (Blue_vs_White) in WT and Mp*cry*. Genes with a fold change ratio (Blue_vs_white) > 1.5 or <0.67 and a *p* value < 0.05 as deferentially expressed genes (DEGs). (**C**) GO enrichment analysis of 1084 DRGs between WT and Mp*cry*.

The Citrine-tagged version of MpCRY protein was localized to the nucleus and cytoplasm.

(Figure S3A,B), implying its involvement in the transcriptional regulation of BL-responsive genes as reported for A. thaliana [47,48,49]. Since Mp*cry* and WT both grew symmetrically under WL but presented partial asymmetric growth under BL, we undertook transcriptome analysis on Mp*cry-7* and WT gemmalings grown under WL and BL (30 μmol m^−2^ s^−1^). In order to compare the transcriptome differences of different genotypes under different light treatments, we calculated a correlation matrix. The Pearson correlation coefficients between the three replicates of each material under each treatment is above 0.98, indicating the repeatability of our experiment. The Pearson correlation coefficients ranged from 0.92 to 0.95 between the three replicates of WT grown in WL and the three replicates of WT grown in BL. Nevertheless, the correlation coefficients decreased (R^2^ = 0.81–0.88) between the three replicates of Mp*cry* grown in WL and the three replicates of Mp*cry* grown in BL (Figure S4A). Therefore, compared to the gene transcription levels in WT, the gene transcription levels in Mp*cry* change more from WL to pure BL (30 μmol m^−2^ s^−1^) irradiation. We also detected a major change in global gene expression, using principal component analysis (PCA). PCA showed that the contribution ratios of PC1 and PC2 were 44.4% and 20.5%, respectively (Figure 3A). Samples of different materials distributed differentially on PC1 and PC2 under different treatment conditions, certifying the diversity of the experimental materials and the variability of experimental treatment conditions. Next, we analysed the expression patterns of shared identified genes in different materals under different light treatments, and found that there are different changes in the expression patterns of WT and Mp*cry* mutant genes under white light and blue light (Figure S4B). We next defined the genes with the expression fold change ratio (BL vs. WL) > 1.5 or <0.67 and *p* value < 0.05 as different expression genes (DEGs). We identified 900 down-regulated genes and 968 up-regulated genes under BL compared with WL in WT, and 990 down-regulated genes and 1116 up-regulated genes in Mp*cry* (Figure 3B). Among the genes down-regulated in Mp*cry*, 340 genes did not show significant expression changes, while 18 genes were up-regulated in WT; among genes up-regulated in Mp*cry*, 294 genes did not show significant expression changes, while 38 genes were down-regulated in WT (Figure 3B). 230 down-regulated genes and 166 up-regulated genes in WT did not respond to BL (30 μmol m^−2^ s^−1^) in Mp*cry* (Figure 3B). There are 1086 differential response genes (DRGs) between WT and Mp*cry* mutants. Subsequently, we undertook GO enrichment analysis on these 1086 differential response genes between WT and Mp*cry* (Table S1). As shown in Figure 3C, the function enrichment of biological processes is mainly concentrated on photosynthesis, the carbohydrate biosynthetic process, transition metal ion transport and response to light stimulus. In Arabidopsis, cryptochromes regulate transcription factor activities, in a direct or indirect way, to regulate the transcriptional level of a myriad of genes induced by BL [9,11,50,51]. Using the website of PlantTFDB, we predicted 18 transcription factor genes possessing significantly over-represented targets in the 1086 DRGs (Table 1), including MpHY5 (Mp1g16800), MpPIF (Mp3g17350) and MpTCP1 (Mp7g09490).

### 3.4. Mpcry Regulates Transcription of Photosynthesis-Related Genes under Blue Light

**Figure 4:**
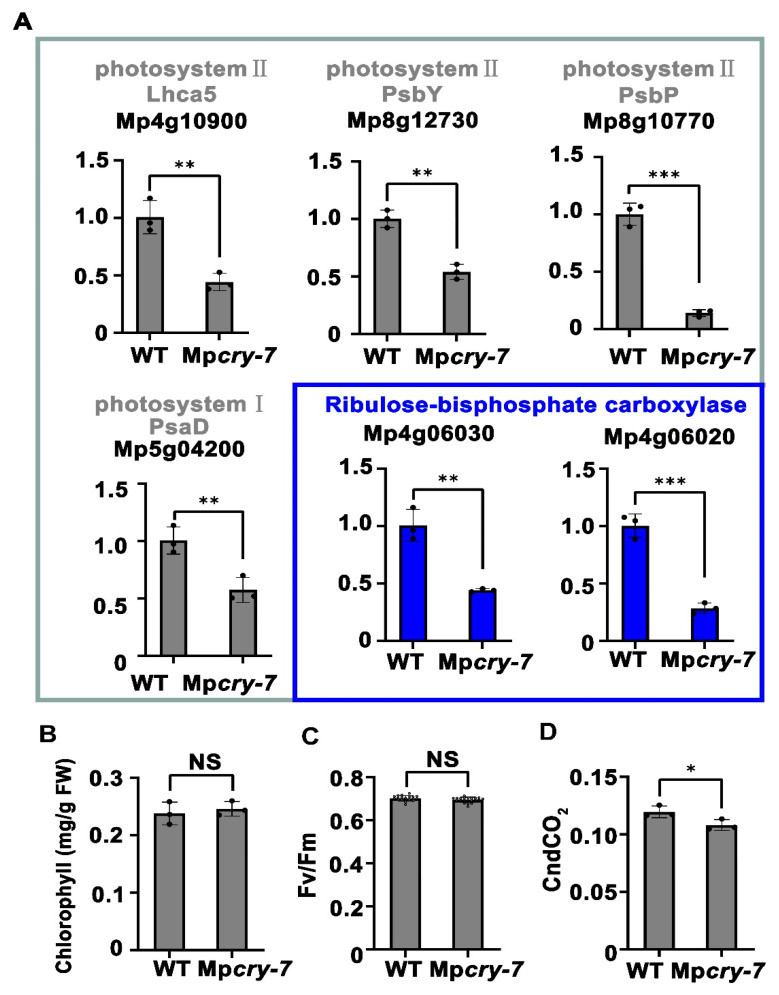
Mp*cry* regulates the transcription of photosynthesis-related genes under BL. (**A**) qPCR showing that the expression of genes coding proteins in photosystem II, photosystem I and ribulose-bisphosphate carboxylase in WT and Mp*cry-7* (*n* = 3 biological replicates). Student’s *t* test: *** *p* < 0.001, ** *p* < 0.01, * *p* < 0.05. (**B**–**D**) Chlorophyll levels (mg/g FW), Fv/Fm and total conductance to CO_2_ for WT and Mp*cry-7* grown under BL. NS, No significance. In (**C**), asterisk indicates *p* < 0.05.

To study the regulatory role of Mp*cry* on photosynthesis, we assessed the photosystems in WT and Mp*cry-7* mutant plants under BL (30 μmol m^−2^ s^−1^) using qPCR. We detected the expression level of putative photosystem I and II component orthologs, including MpLHCA5, an ortholog of light-harvesting complex II chlorophyll a/b binding protein 5 (Mp4g10900), putative photosystem II components MpPsbY (Mp8g12730) and MpPsbP (Mp8g10770), and a putative photosystem I component MpPsaD (Mp5g04200). We also assessed the expression levels of two putative ribulose-bisphosphate carboxylase small subunit genes involved in carbon fixation in photosynthesis, Mp4g06030 and Mp4g06020. The qPCR analysis showed that the expression level of these genes in Mp*cry* mutant plants was significantly lower than that in WT (Figure 4A). Although there was no significant difference in chlorophyll content and maximum quantum efficiency of PSII (Fv/Fm) between WT and Mp*cry* under BL (Figure 4B,C), the absorption efficiency of CO_2_, commonly used to indicate photosynthetic carbon fixation, decreased in Mp*cry* when compared with that in WT (Figure 4D). These results indicate that Mp*cry* induce the expression level of genes involved in photosystem and carbon fixation and Mp*cry* promotes carbon fixation in *M. polymorpha*.

### 3.5. Sucrose Could Partially Restored the Symmetric Growth of the Mpcry Mutant Thallus

**Figure 5:**
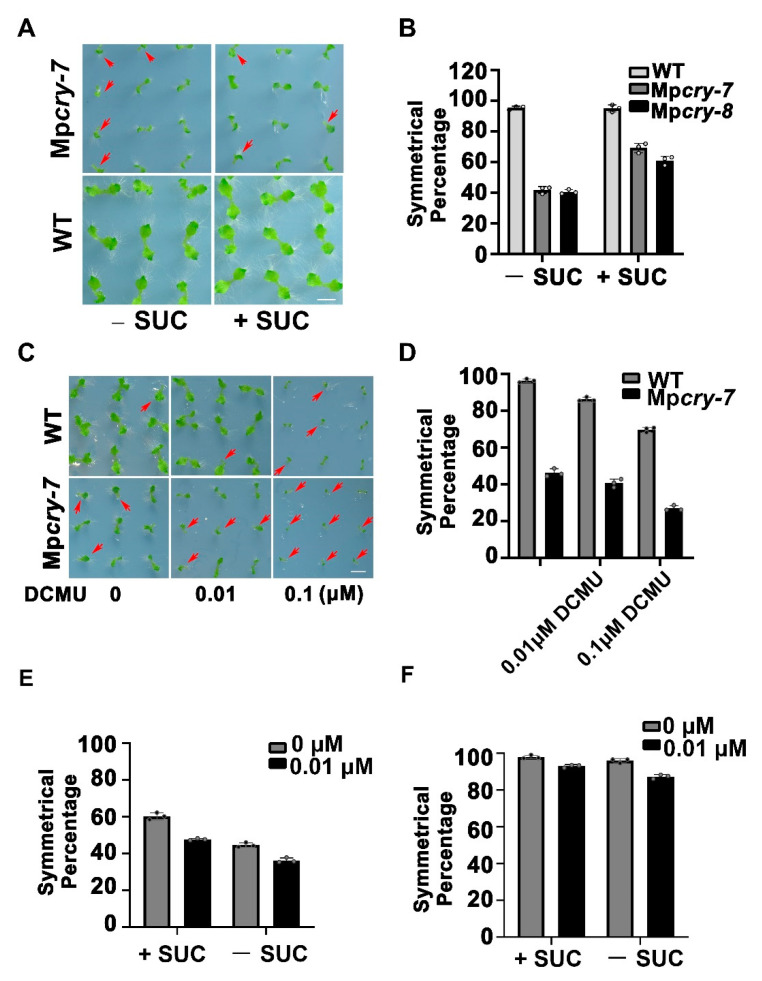
(**A**) Photographs of gemmalings treated without or with 1% sucrose in half-strength Gamborg’s B5 medium under BL (30 μmol m^−2^ s^−1^) for 14 days. Bar = 5 mm. The red arrows represent individuals with asymmetric growth of thalli. (**B**) The symmetrical percentage in (**A**). Data are presented as mean ± SD (*n* = 3 biological statistics, small black circles). More than 100 gemmalings were used for one count. (**C**) Photographs of gemmalings treated with different concentrations of DCMU in half-strength Gamborg’s B5 medium under BL (30 μmol m^−2^ s^−1^) for 14 days. Bar = 5 mm. The red arrows represent individuals with asymmetric growth of thalli. (**D**) The symmetrical percentage in (**C**). Data are presented as mean ± SD (*n* = 3 biological statistics, small black circles). More than 100 gemmalings were used for one count. (**E**,**F**) The symmetrical percentage of symmetrically grown gemmalings in Mp*cry* (**E**) and in WT (**F**). Gemmae were planted on the indicate culture medium under BL (30 μmol m^−2^ s^−1^) for 5 days. +SUC and −SUC represent the culture medium with or without 1% sucrose. Black and Grey column represent the culture medium with and without 0.01 μM DCMU respectively. Data are presented as mean ± SD (*n* = 3 biological statistics, small black circles). More than 100 gemmalings were used for one count.

To verify whether the asymmetric growth of thalli in Mp*cry* is mediated by photosynthesis under BL (30 μmol m^−2^ s^−1^), we provided sucrose to Mp*cry* mutant plants. The percentage of Mp*cry* mutant plants with symmetric thalli raised significantly (Figure 5A,B), suggesting that sucrose plays a supportive role in the symmetric growth of *M. polymorpha* thalli. To test whether defective photosynthetic light reaction leads to the asymmetric growth of thalli, we added photosynthetic inhibitor DCMU with different concentrations into the culture medium. The results showed that, under BL, 0.01 μM DCMU slightly decreased the percentage of symmetrically grown thalli to 86.3% in WT and to 40.7% in Mp*cry*, while 0.1 M DCMU remarkably decreased the symmetrical percentage of thalli to 69.8% in WT and to 27% in Mp*cry* (Figure 5C,D). Besides, DCMU decreases the symmetrical percentage of thalli under WL in WT and Mp*cry*, as under BL (Figure S5A,B), indicating that the inhibition of photosynthetic light reaction of *M. polymorpha* leads to a higher percentage of asymmetric growth on thalli irrespective of light quality. These results suggest that BL and sucrose availability are cooperatively involved in the symmetric growth of *M. polymorpha* thalli, while photosynthetic light reaction influences the symmetry of the thallus growth in a parallel pathway to these two stimuli. To confirm this suggestion, we planted gemmae from Mp*cry* and WT on the culture medium with 0.01 μM DCMU and 1% sucrose. Consistent with this suggestion, the decreased percentage of symmetrically grown thalli affected by DCMU was partially restored (47.8% in Mp*cry* and 92.9% in WT) by sucrose (Figure 5E,F and Figure S5C).

### 3.6. Mpcry Is Likely to Regulate the Symmetric Growth of Thallus through Sucrose Metabolism

**Figure 6:**
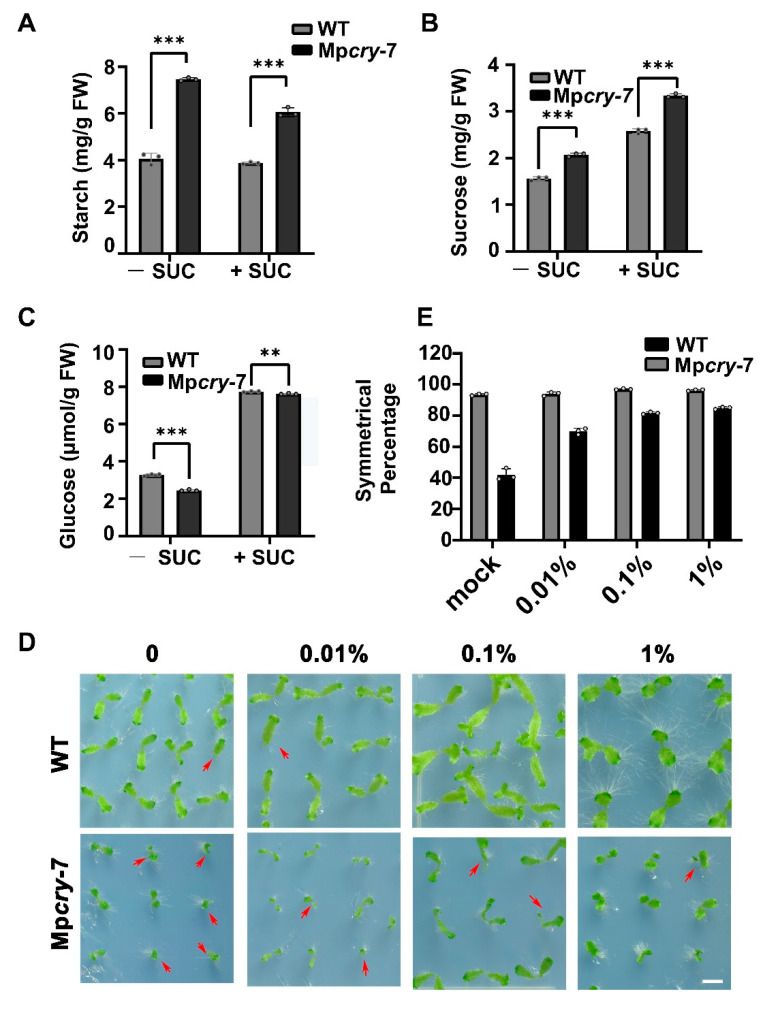
Mpcry regulates the symmetric growth of thalli by affecting glucose content under BL. (**A**–**C**) Quantifications of starch, sucrose, glucose contents in WT and Mp*cry-7* grown under continuous BL for 14 days. Data are presented as mean ± SD (*n* = 3 biological replicates). Student’s *t* test: *** *p* < 0.001, ** *p* < 0.01. (**D**) Photographs of gemmalings treated with different concentrations of glucose under BL (30 μmol m^−2^ s^−1^) for 14 days. Bar = 5 mm. The red arrows represent individuals with asymmetric growth of thalli. (**E**) The percentage of individuals with symmetric growth of thalli in (**D**). Data are presented as mean ± SD (*n* = 3 biological statistics). More than 100 gemmalings were used for one count.

As the photosynthesis activity of Mp*cry* is lower than that of WT, we speculated that the photosynthetic products in Mp*cry* fewer than WT. Interestingly, the content of starch and sucrose in Mp*cry* was significantly higher than that in WT (Figure 6A,B). Therefore, we used qPCR to test if expressions of the sugar metabolism pathway genes are changed in Mp*cry* mutant plants. The results showed that the expression level of a gene encoding putative sucrose-phosphate synthase (SPS; Mp3g02650, Mp2g16990), a key regulator of sucrose biosynthesis, in Mp*cry* was lower than that in WT, and the expression levels of genes encoding sucrose degradation enzymes, invertases (INVs; Mp7g02630, Mp7g01300, Mp4g16290, Mp7g01660, Mp3g02080) and a sucrose synthase (SuS or SuSy; Mp2g16060), in Mp*cry* were significantly reduced compared with those in WT (Figure S6). Consistent with these qPCR results, the content of glucose in Mp*cry* was lower than that in WT (Figure 6C). We applied different amounts of glucose in the culture medium to observe the growth of thalli. The results showed that exogenous application of 0.01% glucose could significantly increase the symmetrical percentage of thalli in Mp*cry* (from 42% to 69.9%), while the application of a higher concentration of glucose (0.1% and 1%) did not raise the percentage of symmetrical growth in Mp*cry* thalli (81.6% and 84.8%, respectively) (Figure 6D,E). These results suggest that glucose availability regulates the symmetric growth of thalli in *M. polymorpha*.

## 4. Discussion

Energy provided from light is the basis for the normal growth and development of plants. Plants have evolved complex photoreceptor systems to maximize photosynthetic products and make better use of photosynthetic products [52,53]. A previous study showed that the photosynthesis efficiency of Arabidopsis *cry1cry2* mutants was reduced [54]. The mechanism of cryptochrome regulating photosynthesis has also been studied. *Arabidopsis* CRY1 induces the expression of *SIG5* to promote the transcription of genes encoding photosystem II core proteins psbA and pcbD under BL [55,56,57]. *Arabidopsis* CRY2 induces *ATAB2* to regulate the photosystem formation of PSI and PSII [58]. Previous transcription and proteomics experiments speculated that CRY2 can affect tomato photosynthesis and the biosynthesis of starch and sucrose [59]. In addition, a recent study showed that tomato CRY1a can promote starch degradation [60]. These studies suggest that cryptochromes play an important role in the regulation of photosynthesis and the utilization of photosynthetic products in vascular plants. However, it has not been studied whether cryptochromes still have these function in bryophytes. In this study, we showed the roles of cryptochrome in carbon fixation and sucrose metabolism in *M. polymorpha*.

Previous studies have shown that a mixture of RL and far-red light to certain scale significantly affect the germination of gemmae and the morphology of thalli [46]. In this study, we confirmed that BL was associated with the slender thalli and oblique angle growth (Figure S1), and asymmetric growth of thalli in *M. polymorpha* (Figure 2C,D). Such asymmetry on thalli was actually caused by the asymmetric germination of gemmae in *M. polymorpha* (Figure S2). In addition, we used transcriptome analysis and found that Mp*cry* mainly regulates transcription of genes for photosynthesis and sugar metabolism (Figure 3C), suggesting the contribution of Mp*cry* to photosynthesis and utilization of photosynthetic products in *M. polymorpha*. Then we found that the efficiency of carbon fixation in Mp*cry* was lower than that in WT and that sucrose increased the percentage of individuals with asymmetrical growth of thalli in Mp*cry* (Figure 5A,B), indicating MpCRY positively regulates carbon fixation. Although the transcription of genes encoding proteins in PSII and PSI was down regulated in Mp*cry*, the maximum quantum yield of photochemical products in PSII did not significantly decrease. These findings suggest that the photosystems may be tolerant to overall down-regulation of most photosynthesis-related genes, but carbon fixation and metabolism may not be. A previous study showed that loss of Photosynthesis-Related RAF (MpPRAF) impaired carbon metabolism but did not have any significant impact on Fv/Fm value [61]. However, MpPRAF is not included in the DEGs of WT and Mp*cry*. Further experiments are needed to prove whether MpCRY regulates the protein level of MpPRAF. In addition, we found both WT and Mp*cry* mutants showed an increase in asymmetric growth by DCMU treatment under WL or BL. This result indicates that reduced photosynthesis capacity in *M. polymorpha* can increase the percentage of individuals with asymmetrical thalli. The decreased percentage of symmetrically grown thalli affected by DCMU was partially restored by sucrose, indicating that photosynthetic light reaction and sucrose influence the symmetry of the thallus growth in a parallel pathway.

Transcriptome analysis also showed 18 transcription factor genes possessing significantly over-represented targets in the 1086 DRPs (Table 1). As these transcriptional factors, such as HY5, have been proven to be regulated by cryptochromes in Arabidopsis [11,50,51], it is reasonable to speculate that cryptochromes play conserved roles in the regulation of these transcription factors. Recent studies have shown that HY5 is a key factor transmitting the nuclear signal, which is mediated by the blue light receptor cryptochromes, to chloroplasts [62]. HY5 not only regulates photosynthesis, but also starch degradation [60,63]. So, whether MpCRY regulates photosynthesis and carbon allocation through HY5 needs further study.

In Mp*cry*, the efficiency of carbon fixation was lower compared with that in WT, but the content of starch and sucrose was higher (Figure 6A,B). Our study showed that the expression of invertases was down-regulated in Mp*cry* (Figure S5B). Generally, invertases decompose sucrose into glucose and fructose [64], and glucose can induce the germination of *M. polymorpha* spores in the dark [45]. These studies suggest a critical role of glucose in the growth and development in *M. polymorpha*. Consistently, our study showed that symmetric growth of Mp*cry* thalli was restored when glucose was added into the culture medium (Figure 6D). Taken together, we speculate that the reduction of carbon fixation in Mp*cry* affects the overall carbon level and that the decrease of sucrose degradation reduces the content of glucose, thus contributing to the asymmetric germination of gemmae. More experiments need to be performed to proof this speculation. For example, whether the knock-out mutants of genes encoding sucrose degradation enzymes also exhibit increased percentage of asymmetric growth of thalli and whether overexpressed MpCRY in these knock-out mutants can restore this phenotype. In addition, we also speculate that there seems to be a mechanism to concentrate glucose or some downstream substances of glucose to one meristem when its availability is limited. It would be interesting to study this mechanism.

## Figures and Tables

**Table 1:** 18 transcription factor genes possessing significantly over-represented targets in the 1086 DRGs.

TF	Background All	Background _Bind	Query_All	Query_Bind	*p*_Value	q_Value	Best Hit in *Arabidopsis thaliana*	Description
Mp7g09490	19,287	404	1086	43	2.19 × 10^−5^	1.78 × 10^−3^	AT1G69690	TCP family protein
Mp8g03820	19,287	490	1086	48	7.06 × 10^−5^	2.53 × 10^−3^	AT5G28770	bZIP family protein
Mp2g23600	19,287	604	1086	56	9.36 × 10^−5^	2.53 × 10^−3^	AT1G49720	abscisic acid responsive element-binding factor 1
Mp6g03920	19,287	484	1086	41	3.64 × 10^−3^	7.38 × 10^−2^	AT1G75390	basic leucine-zipper 44
Mp6g18650	19,287	571	1086	45	9.36 × 10^−3^	1.52 × 10^−1^	AT5G19790	related to AP2 11
Mp1g13640	19,287	103	1086	11	1.30 × 10^−2^	1.57 × 10^−1^	AT1G20980	squamosa promoter binding protein-like 14
Mp5g06970	19,287	436	1086	35	1.41 × 10^−2^	1.57 × 10^−1^	AT5G13910	ERF family protein
Mp2g07170	19,287	503	1086	39	1.77 × 10^−2^	1.57 × 10^−1^	AT2G46270	G-box binding factor 3
Mp5g12480	19,287	520	1086	40	1.88 × 10^−2^	1.57 × 10^−1^	AT1G72360	ERF family protein
Mp4g14530	19,287	162	1086	15	2.06 × 10^−2^	1.57 × 10^−1^	AT1G66810	C3H family protein
Mp5g21080	19,287	1391	1086	95	2.13 × 10^−2^	1.57 × 10^−1^	AT5G63090	LBD family protein
Mp6g04830	19,287	239	1086	20	2.92 × 10^−2^	1.70 × 10^−1^	AT3G49690	myb domain protein 84
Mp2g02230	19,287	415	1086	32	2.92 × 10^−2^	1.70 × 10^−1^	AT2G40950	bZIP family protein
Mp2g13020	19,287	225	1086	19	2.94 × 10^−2^	1.70 × 10^−1^	AT5G08520	MYB family protein
Mp3g17350	19,287	359	1086	28	3.27 × 10^−2^	1.77 × 10^−1^	AT1G09530	phytochrome interacting factor 3
Mp7g02640	19,287	274	1086	22	3.67 × 10^−2^	1.86 × 10^−1^	AT3G20770	EIL family protein
Mp1g25020	19,287	3001	1086	189	4.00 × 10^−2^	1.91 × 10^−1^	AT1G72050	transcription factor IIIA
Mp1g16800	19,287	400	1086	30	4.51 × 10^−2^	2.03 × 10^−1^	AT5G11260	bZIP family protein

## Data Availability

RNA-Seq data in this study had been submitted to NCBI with the SRA accession of PRJNA754679.

## Supplementary Materials

The following are available online at https://www.mdpi.com/article/10.3390/cells10123387/s1, Figure S1: Appearance of the top and side of the thalli under the indicated light conditions; Figure S2: Photographs of WT and Mp*cry*-7 gemmae of *M. polymorpha*; Figure S3: Confocal microscopy images of Citrine; bright-field and merged images of *pro35S::MpCRY*-*Citrine* and pro*35S::MpCRY-Tdtomato* transgenic lines; Figure S4: Transcriptome differences of different genotypes under different light treatments; Figure S5: DCMU inhibits the symmetrical growth of thalli under WL; Figure S6: Mp*cry* mutants affect expression of genes involved in sucrose metabolism, Table S1: Go enrichment. Table S2: Primers in this study.
