# Supplementary material for: Roles of a Cryptochrome in Carbon Fixation and Sucrose Metabolism in the Liverwort Marchantia polymorpha
Source: Cells. 2021 Dec 1;10(12):3387. doi: 10.3390/cells10123387 (PMC8699372; doi:10.3390/cells10123387)
Supplement: Supplementary file 1 [file cells-10-03387-s001.zip › Revised Supplementary figures.pdf]

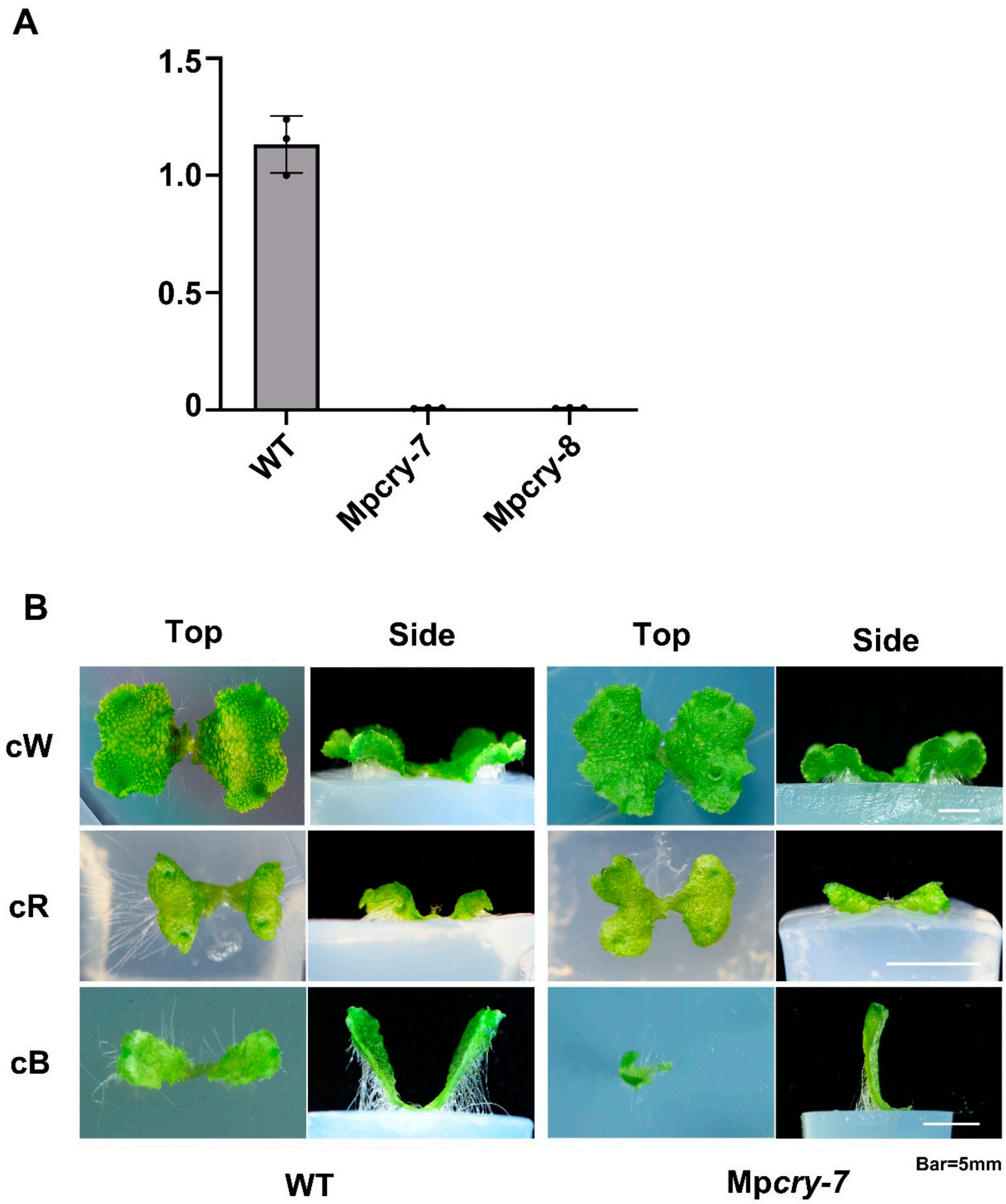

Figure S1 Appearance of the top and side of the thalli under the indicated light conditions. (A) The mRNA levels of *MpCRY* in WT, Mp*cry-7* and Mp*cry-8* mutants. Data are presented as mean ± SD (n = 3 biological replicates). (B) The gemmalings of WT and Mp*cry-7* were grown under continuous WL (WL; 50 μmol m^{-2} s^{-1}), continuous RL (RL; 30 μmol m^{-2} s^{-1}) or continuous BL (BL; 30 μmol m^{-2} s^{-1}) for 14 days, respectively. Bar = 5mm.

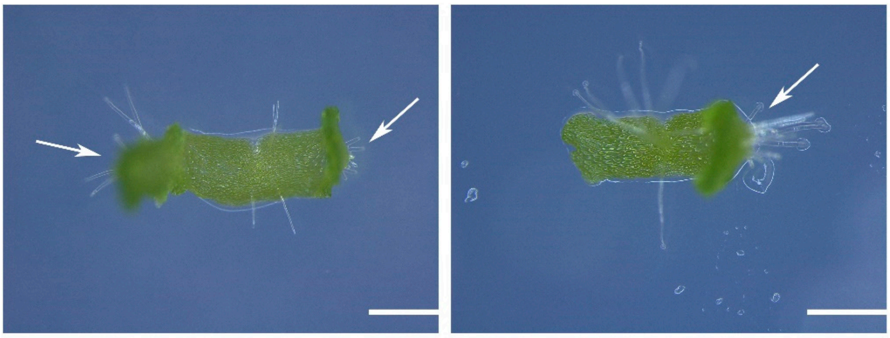

WT **Mp*cry*-7**

Figure S2 Photographs of WT and Mp*cry*-7 gemmae of *M. polymorpha*.
Symmetrical germinated (WT, left panel) and asymmetrical germinated (right panel) gemmae are shown. Bar = 1 mm. The white arrows represent the gemmae of germination.

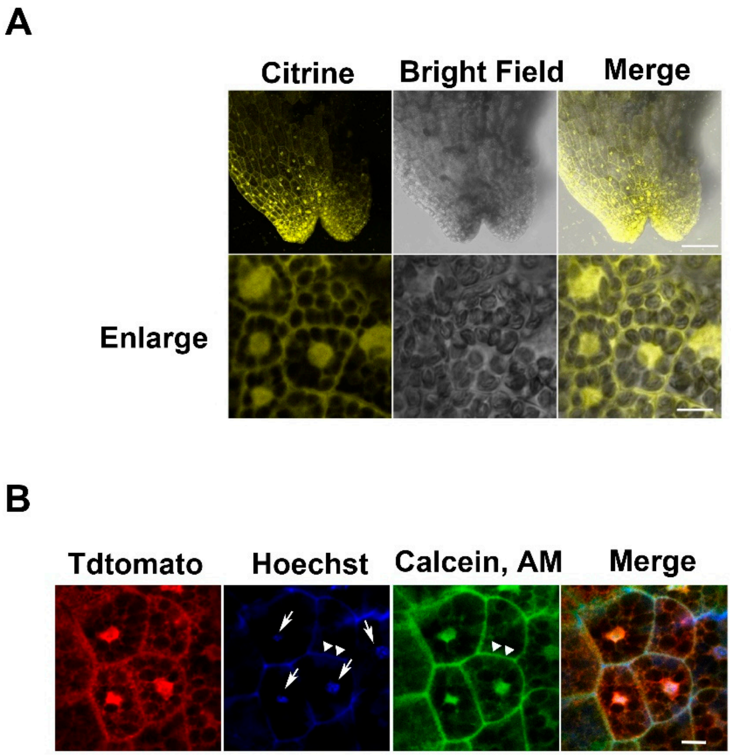

Figure S3 Confocal microscopy images of Citrine; bright-field and merged images of *pro35S::MpCRY-Citrine* and pro*35S::MpCRY-Tdtomato* transgenic lines.

(**A**) 3-d-old gemmaling expressing *pro35S::MpCRY-Citrine* was observed. Bar = 100 µm in top; 10 µm in down. Similar results were observed in 3 independent repeats. (**B**) 3-d-old gemmaling expressing pro*35S::MpCRY-Tdtomato* was observed. The white arrows represent the nucleus. The white arrow heads represent the dyes that have not penetrated into the cell. Bar = 5 µm. Similar results were observed in 3 independent repeats.

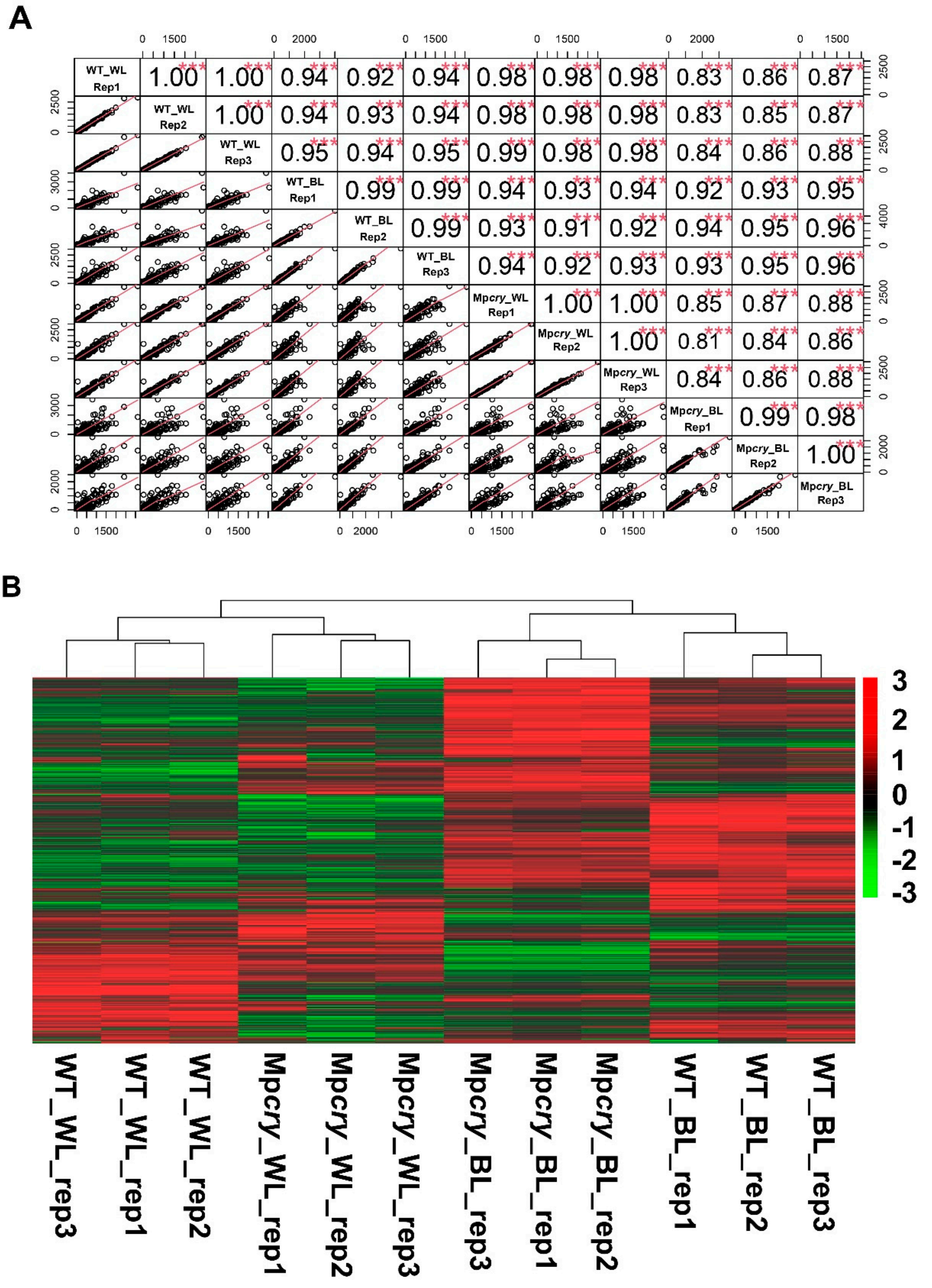

Figure S4 Transcriptome differences of different genotypes under different light treatments. (**A**) Pearson correlation matrix showing the correlation between three replicates in different genotypes under different treatments. Three stars mean that the corresponding variable is significant at a 1% level. (**B**) Heatmap of shared identified genes in three replicates from different genotypes under different treatments.

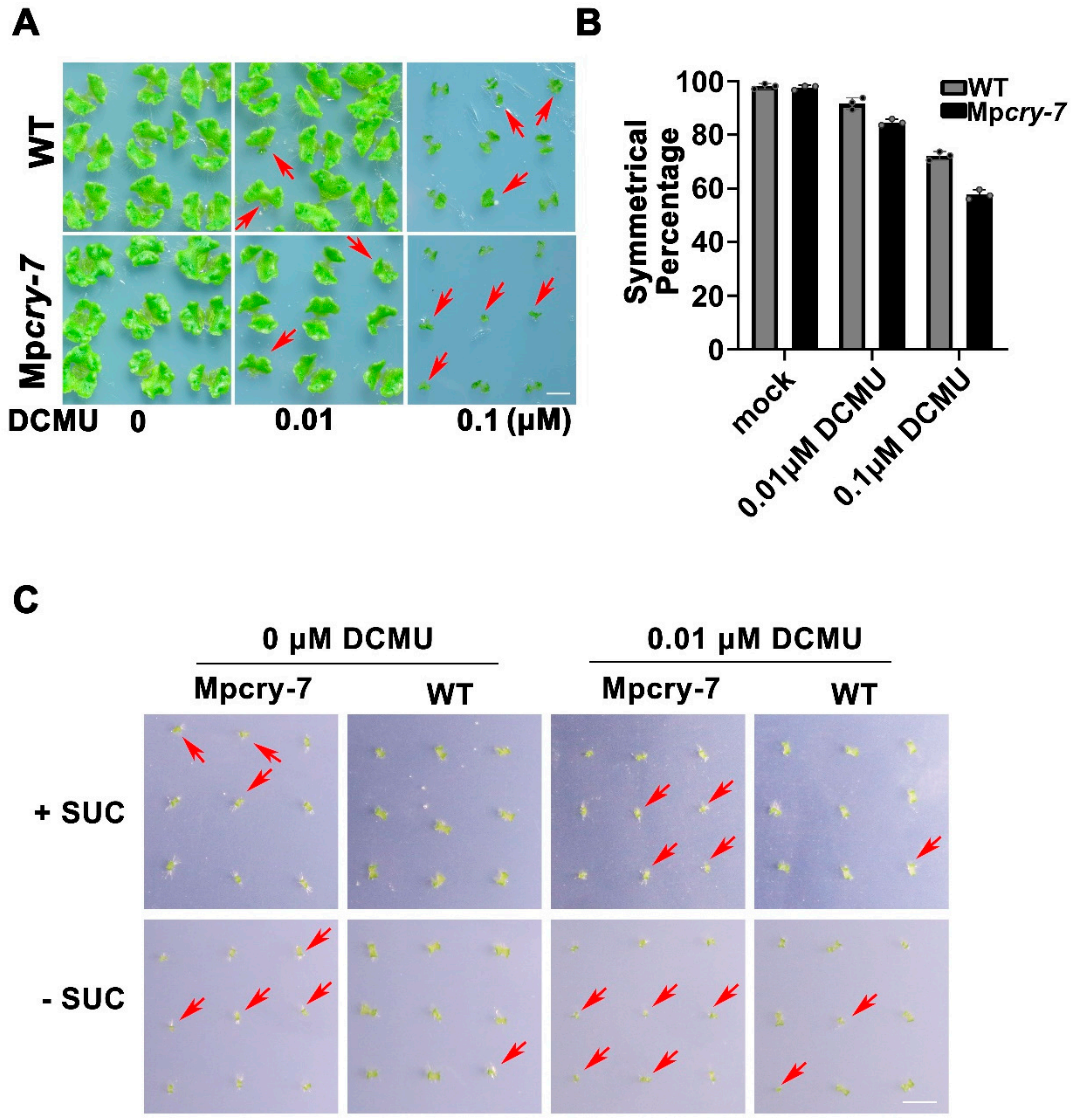

Figure S5 DCMU inhibits the symmetrical growth of thalli under WL.

(**A**) Photographs of gemmalings treated with different concentrations of DCMU in half-strength Gamborg's B5 medium under WL (WL; 50 μmol m^{-2} s^{-1}) for 14 days. Bar = 5 mm. The red arrows represent individuals with asymmetrical growth of thalli. (**B**) The percentage of individuals with symmetrical growth of thalli in (**A**). Data are presented as mean ± SD (n=3 biological statistics). More than 100 gemmalings were used for one count. (**C**) Photographs of gemmalings planted on indicated culture medium under blue light for 5 days. Bar = 5 mm. The red arrows represent individuals with asymmetrical growth of gemmalings.

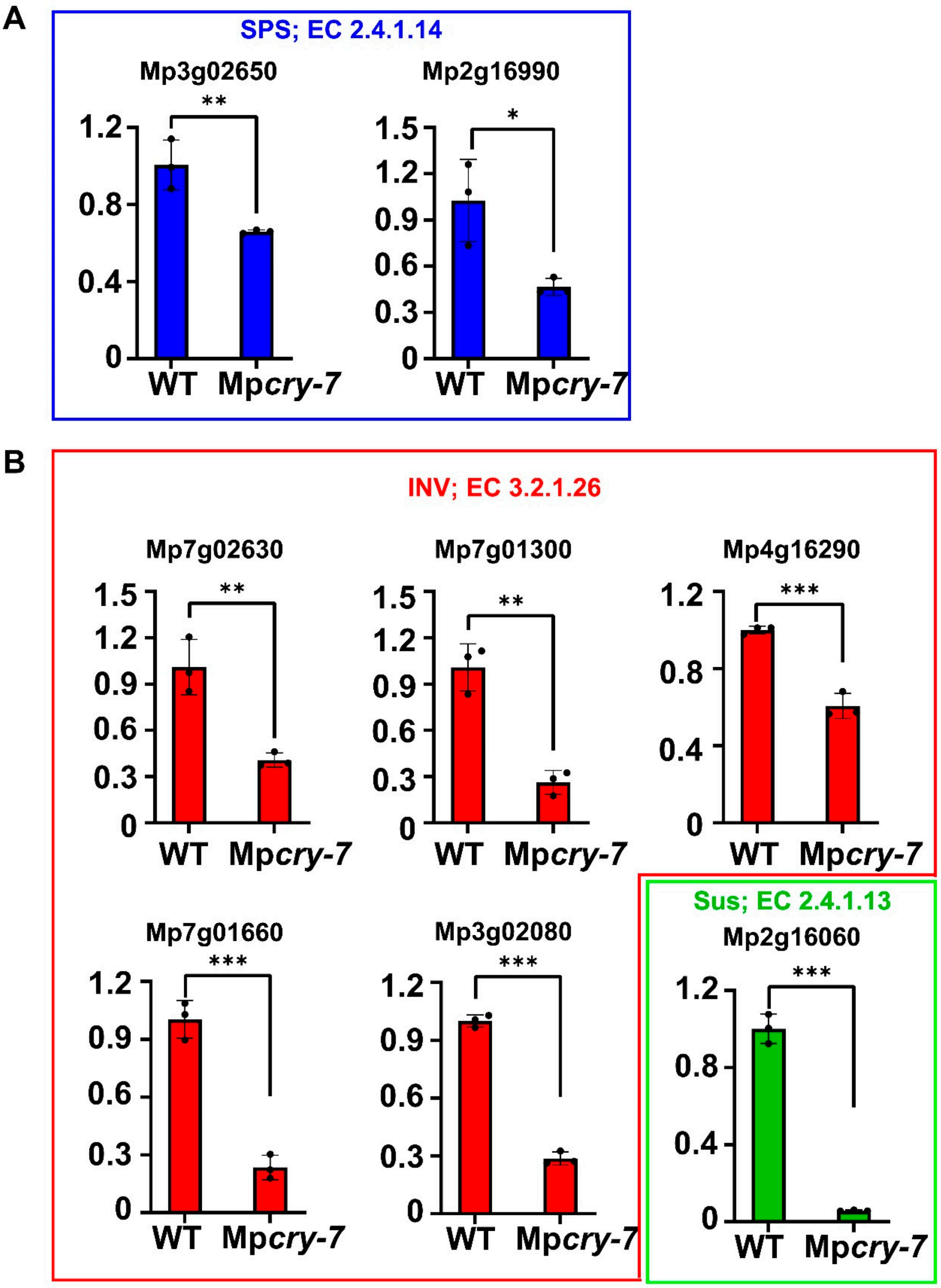

Figure S6 Mp*cry* mutants affect expression of genes involved in sucrose metabolism.

(**A**) Expression of genes coding Suc-phosphate synthase in WT and Mp*cry*-7 (n = 3 biological replicates). (**B**) Expression of genes coding invertase and Suc synthase in WT and Mp*cry*-7 (n = 3 biological replicates).
